# Editorials

**Published:** 1895-04

**Authors:** 


### EDITORIAL.

### Important Notice.

The Journal wishes to give notice to all of its subscribers that, beginning with the March number, the publishers will refuse to replace any numbers lost in transmission through the mails, unless a claim is filed for the same within thirty days from the date of mailing from the publication office. Beginning with the April number the Journal will be regularly sent out between the 5th and 10th of each month. We do this because it is next to impossible to trace up Journals lost for two or three months and to place the proper responsibility for non-delivery.

W. Horace Hoskins,
Business Manager.

3452 Ludlow Street, Philadelphia.

We print in this number a very interesting and suggestive letter from the pen of Dr. F. M. Perry on the subject of the most comprehensive and uniform degree for the veterinarian. As this subject is one of great importance and merit to the profession, and as it is still one of the vexatious and unsolved questions before the Association of Veterinary Faculties of North America, the Journal will offer its columns for the next few months for the publication of short letters and opinions bearing on this subject.

Since the experimental stage is already past we think it is well for the public in general to consider the fact that no cities or States should enter into the manufacture of antitoxin or any other drug, but should leave its production, as all other commodities should be, in the hands of the people. Money has already been appropriated by state and local governments where the same was to be used for charitable purposes and in behalf of the poor, and were justified only on the grounds that they would thus be assured of a proper preparation, but it is always a dangerous precedent, and is never supported by public approval, for National, State or local governments to be engaged in the manufacture of productions in competition with the people.

